# Remarkable Response to Radiotherapy With Simultaneous Integrated Boost Intensity-Modulated Radiotherapy for Locally Advanced Lymphoepithelial Carcinoma of the Hypopharynx in a Nonagenarian

**DOI:** 10.7759/cureus.79939

**Published:** 2025-03-02

**Authors:** Atsuto Katano, Subaru Sawayanagi, Hideomi Yamashita

**Affiliations:** 1 Radiology, The University of Tokyo Hospital, Tokyo, JPN

**Keywords:** elderly patients, head and neck neoplasm, hypopharyngeal cancer, intensity-modulated radiotherapy, lymphoepithelial carcinoma, radiotherapy, simultaneous integrated boost

## Abstract

Lymphoepithelial carcinoma of the head and neck is a rare malignant neoplasm, with primary involvement of the hypopharynx being exceptionally uncommon. While surgery remains the standard treatment, radiotherapy plays a crucial role, particularly for patients who are not surgical candidates. Recent advancements in radiotherapy, such as simultaneous integrated boost (SIB) intensity-modulated radiotherapy, have demonstrated promising outcomes with minimal invasiveness, making them a viable option, especially for elderly patients. This case report describes a 91-year-old female diagnosed with locally advanced lymphoepithelial carcinoma of the hypopharynx who was treated with radiotherapy using SIB intensity-modulated radiotherapy. The patient presented with dysphagia and was found to have a large hypopharyngeal mass with nodal metastasis. Biopsy and immunohistochemical analysis confirmed the diagnosis of lymphoepithelial carcinoma. Given her age and comorbidities, a multidisciplinary team opted for radiotherapy instead of surgery. The prescribed dose was delivered using helical tomotherapy with two gradient doses of 30 gray (Gy) and 35 Gy in 10 fractions utilizing the SIB method. Acute toxicities were mild, and the patient completed the treatment without interruption. Post-treatment imaging revealed a remarkable response, which was sustained for over one year without disease progression.

## Introduction

Lymphoepithelial carcinoma (LEC) of the head and neck is a rare malignant neoplasm, accounting for less than 1% of all salivary gland malignancies [1]. The most common location of LEC of the head and neck is the salivary glands, particularly the parotid gland. While surgery remains the cornerstone of treatment, radiotherapy also plays a significant role as an adjuvant therapy, similar to other parotid-origin malignancies [2].

However, LEC may arise at other sites, including the oropharynx, sinonasal tract, larynx, tongue, and temporal bone [3,4]. LEC of the salivary glands and nasopharynx is frequently associated with Epstein-Barr virus (EBV), whereas its involvement in laryngeal and hypopharyngeal cases is uncommon [5]. LEC of the hypopharynx and larynx is exceptionally rare, with Faisal et al. reporting fewer than 50 documented cases in the literature [6]. Their review, which analyzed 21 studies involving 46 patients, emphasized key clinical features, including frequent involvement of the supraglottis and pyriform sinus, male predominance, and predominance of surgery combined with adjuvant therapy as the primary treatment approach. In certain cases, radiotherapy may provide sufficient efficacy as a stand-alone modality or in combination with other treatments, particularly for patients who are not surgical candidates.

Recent advancements in radiotherapy have yielded excellent outcomes with minimal invasiveness, making it a viable option for elderly patients [7-9]. This report highlights a rare case of LEC of the hypopharynx diagnosed at an advanced stage. Conventional palliative radiotherapy, typically delivered as 30 Gy in 10 fractions, is associated with a median survival of approximately 7.5 months [10]. In this case, the patient received radiotherapy using the simultaneous integrated boost (SIB) technique, which allowed for a more efficient dose escalation to the tumor while maintaining the overall treatment duration. This approach aimed to achieve better long-term disease control. The treatment resulted in a remarkable response, which was sustained for over one year without moderate-to-severe adverse events.

## Case presentation

A 91-year-old female presented to her primary care physician with a chief complaint of dysphagia, persisting for three months. Upper gastrointestinal endoscopy revealed a deformity with an elevated lesion in the right hypopharynx, for which she was referred to our hospital. The patient had no history of alcohol consumption or smoking. Her medical history included bronchial asthma managed with inhalers, hypertension under treatment, and a lumbar disc herniation. The laboratory data showed the following: total protein was 7.8 g/dL (normal range: 6.6-8.1 g/dL), albumin was 3.5 g/dL (normal range: 4.1-5.1 g/dL, low), lactate dehydrogenase was 178 U/L (normal range: 124-222 U/L), white blood cell count was 6.7×10³/μL (normal range: 3.3-8.6×10³/μL), red blood cell count was 3.62×10⁶/μL (normal range: 3.86-4.92×10⁶/μL, low), and platelet count was 38.1×10⁴/μL (normal range: 15.8-34.8×10⁴/μL, high). The Karnofsky performance status was 90.

An endoscopic biopsy of the hypopharyngeal lesion was performed. Histopathological examination of the biopsy specimens revealed findings consistent with LEC. The tumor exhibited a reticular growth pattern of atypical cells with enlarged nuclei, sparse keratinization, and a dense infiltration of lymphocytes and plasma cells. Lymphocytes also infiltrated tumor nests. Immunohistochemical analysis revealed that the tumor cells were positive for cytokeratin AE1/AE3 and negative for p63, p40, cytokeratin 5/6, and Epstein-Barr encoding region in situ hybridization. These findings confirmed the diagnosis of hypopharyngeal LEC.

**Figure 1:**
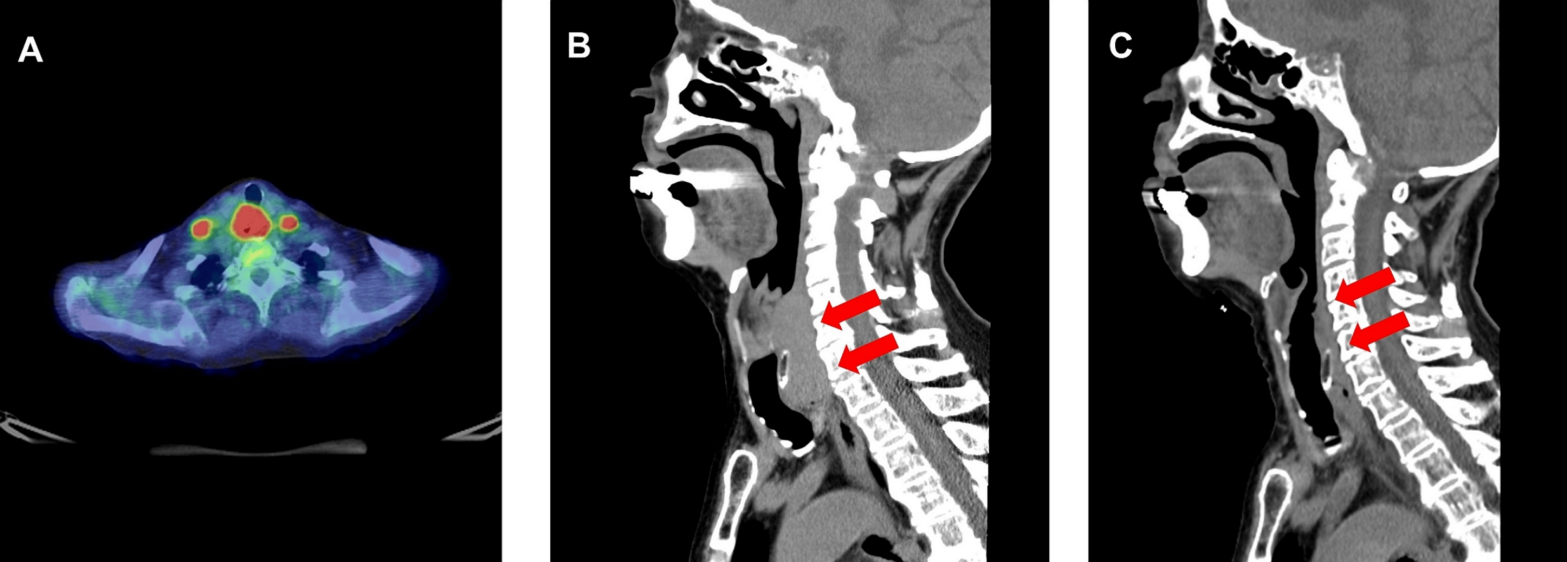
Radiological imaging of lymphoepithelial carcinoma of the hypopharynx in a nonagenarian (A) Pretreatment positron emission tomography-computed tomography (PET/CT) image showing intense fluorodeoxyglucose uptake in the red area of the primary tumor and the involved cervical lymph nodes. (B) Pretreatment sagittal CT scan revealing a hypopharyngeal mass in the cervical region prior to radiotherapy, as indicated by red arrows. (C) A post-treatment sagittal CT scan was performed three months after radiotherapy and demonstrated a marked reduction in the lesion size, indicated by red arrows.

Positron emission tomography-computed tomography (PET/CT) revealed a mass in the hypopharynx with significant fluorodeoxyglucose uptake and a maximum standardized uptake value of 17.28, which was consistent with primary hypopharyngeal carcinoma (Figure 1A).

The enlarged bilateral level VI cervical lymph nodes demonstrated a maximum standardized uptake value of 11.25, indicating nodal metastasis. No evidence of distant metastases or abnormal fluorodeoxyglucose uptake was observed. Computed tomography (CT) identified a 64 mm hypopharyngeal mass involving the lateral and posterior walls from the hyoid level to the superior thyroid cartilage (Figure 1B). The tumor extended beyond the thyrohyoid membrane into the right deep cervical soft tissue without overt destruction of the hyoid bone. At the thyroid cartilage level, invasion of the paraglottic space and posterior cricoid cartilage was noted, with no apparent bone destruction. The mass extended inferiorly into the cervical esophagus and displaced the membranous tracheal wall anteriorly. Enlarged bilateral level VI lymph nodes were evident, consistent with metastatic involvement.

**Figure 2:**
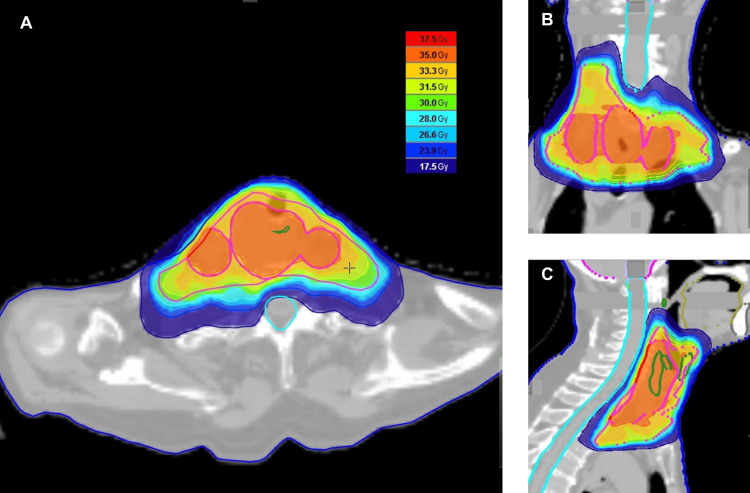
Dose distribution in radiotherapy planning for lymphoepithelial carcinoma of the hypopharynx The radiotherapy dose distribution for lymphoepithelial carcinoma of the hypopharynx demonstrates precise target coverage and sparing of surrounding normal tissues in axial (A), coronal (B), and sagittal (C) views. The sky-blue line indicates the spinal canal. The color scale on the right represents the radiation dose levels in gray (Gy).

The patient was clinically staged as T4aN2cM0 (lymphoepithelial carcinoma) according to the American Joint Committee on Cancer 8th edition staging system. Following a multidisciplinary team discussion, surgery was deemed inappropriate, and a radiotherapeutic approach was chosen. Radiotherapy was administered using helical tomotherapy with SIB, following the standard protocol used at our institution [11]. The patient was immobilized using a thermoplastic mask for radiotherapy. Treatment planning was based on CT images acquired with a slice thickness of 2 mm. The prescribed dose for SIB was set at two gradients, 35 Gy and 30 Gy, over 10 fractions. Gross tumor volume 35 (GTV35) was defined as a primary tumor with lymph node involvement. Clinical target volume 35 (CTV35) was created by isotropically expanding the GTV35 by 5 mm while accounting for the anatomical structures. Based on international guidelines, CTV30 was defined to include left cervical lymph node levels IVa and IV, as well as right cervical lymph node levels II, III, IVa, and VI. The planning target volumes (PTV35 and PTV30) were generated by expanding the CTV35 and CTV30 by 3 mm, respectively, with both volumes cropped to 3 mm from the skin. The radiotherapy dose parameters are shown in Table 1, and the dose distribution of the radiotherapy plan is shown in Figure 2. The conformity index and homogeneity index were 1.085 and 1.066, respectively.

**Table 1:** Dosimetric parameters for target volumes and organs at risk Dosimetric parameters, including volume, maximum dose, minimum dose, median dose, and average dose for the target volumes (PTV35 and PTV30) and critical structures. PTV: planning target volume

Name	Volume (cc)	Maximum Dose (Gy)	Minimum Dose (Gy)	Median Dose (Gy)	Average Dose (Gy)
PTV35	169.43	38.16	33.48	35.8	35.8
PTV30	199.92	36.02	28.15	33.54	33.25
Oral Cavity	71.05	28.57	0.41	1.41	4.04
Spinal Cord	23.8	15.1	0.07	1.5	5.37
Parotid (Left)	21.45	6.69	0.24	0.54	1.05
Parotid (Right)	20.84	9.86	0.31	0.81	1.28
Mandible	65.44	16.93	0.24	3.66	4.56

According to the evaluation based on the Common Terminology Criteria for Adverse Events version 5.0, acute adverse events of radiotherapy included grade 1 mucositis and grade 1 dysphagia. The patient completed the scheduled course of radiotherapy without any interruptions.

Several days after completing radiotherapy, the patient began to notice an improvement in her difficulty swallowing. Three months later, a CT scan revealed a noticeable reduction in the mass, predominantly involving the right wall of the hypopharynx, with no apparent signs of invasion (Figure 1C). Significant shrinkage of the enlarged bilateral level VI lymph nodes was observed. One and a half years after radiotherapy, the disease remained stable without any progression. Only mild grade 1 hoarseness was observed as a late adverse event associated with radiotherapy.

## Discussion

LEC of the hypopharynx is a rare malignancy characterized by dense lymphocytic infiltration and aggressive clinical behavior. Optimal treatment management of LEC using multimodal approaches is often required to achieve disease control.

Qiu et al. aimed to evaluate the survival outcomes in patients with LEC of the major salivary glands treated with either upfront surgery or chemoradiotherapy [12]. Among the 107 patients, the five-year locoregional failure-free and overall survival rates were 86.6% and 84.4%, respectively. They reported that upfront chemoradiotherapy demonstrated effectiveness comparable to that of upfront surgery in terms of locoregional control and overall survival. Niu et al. conducted a retrospective study of 56 patients with LEC of the salivary gland treated with radical radiotherapy, with or without surgery [13]. At a median follow-up of 60 months, the five-year progression-free survival, locoregional progression-free survival, distant metastasis-free survival, and overall survival rates were 84.7%, 87.4%, 86.7%, and 92.4%, respectively, in patients with mild treatment-related toxicities. Lv et al. described three advanced LEC cases treated with induction chemotherapy followed by concurrent chemoradiotherapy, all of which achieved complete responses with no local or regional recurrence over follow-up periods of 6-50 months and without serious adverse events [14].

The SIB technique delivers various dose levels to different target volumes within a single session, optimizing the treatment by preserving the overall treatment time while escalating the radiation dose to the boost volume. Maintaining the overall treatment duration may affect tumor control and normal tissue response, potentially influencing the clinical efficacy of SIB regimens in head and neck cancer [15,16]. SIB may be a promising option for the careful selection of elderly patients. For head and neck tumors in elderly patients, the prognosis with conventional palliative radiotherapy is typically approximately seven months [17]. However, this patient achieved long-term tumor control. Moreover, the favorable response observed in this case underscores that age alone should not preclude radiotherapy, even in nonagenarian patients [18].

## Conclusions

This case highlights the importance of individualized treatment strategies that consider patient preferences, performance status, and life expectancy. A multidisciplinary approach remains essential for managing such complex cases, ensuring the integration of palliative and supportive care with definitive oncologic therapy.

Further research would be beneficial to better understand the broader applicability of SIB in elderly patients with LEC and other aggressive head and neck cancers. Elderly patients with cancer often face multiple, unaddressed challenges in routine clinical care, which can be effectively identified by quantitative assessments such as the comprehensive geriatric assessment. Implementing these assessments can help to address specific issues and improve outcomes. Additionally, exploring the role of molecular and immunological profiling could enhance therapeutic decision-making, particularly for rare cancers such as hypopharyngeal LEC.
